# New insights into the nutritional genomics of adult-onset riboflavin-responsive diseases

**DOI:** 10.1186/s12986-023-00764-x

**Published:** 2023-10-16

**Authors:** Chiara Murgia, Ankush Dehlia, Mark A. Guthridge

**Affiliations:** 1 The School of Agriculture, Food and Ecosystem Sciences (SAFES), Faculty of Science, The University of Melbourne, Parkville, Australia; 2 School of Life and Environmental Sciences, Deakin University, Burwood, Australia

**Keywords:** Nutrigenomics, Micronutrients, Vitamin, Metabolism, Personalised nutrition

## Abstract

Riboflavin, or vitamin B2, is an essential nutrient that serves as a precursor to flavin adenine dinucleotide (FAD) and flavin mononucleotide (FMN). The binding of the FAD and/or FMN cofactors to flavoproteins is critical for regulating their assembly and activity. There are over 90 proteins in the human flavoproteome that regulate a diverse array of biochemical pathways including mitochondrial metabolism, riboflavin transport, ubiquinone and FAD synthesis, antioxidant signalling, one-carbon metabolism, nitric oxide signalling and peroxisome oxidative metabolism. The identification of patients with genetic variants in flavoprotein genes that lead to adult-onset pathologies remains a major diagnostic challenge. However, once identified, many patients with adult-onset inborn errors of metabolism demonstrate remarkable responses to riboflavin therapy. We review the structure:function relationships of mutant flavoproteins and propose new mechanistic insights into adult-onset riboflavin-responsive pathologies and metabolic dysregulations that apply to multiple biochemical pathways. We further address the vexing issue of how the inheritance of genetic variants in flavoprotein genes leads to an adult-onset disease with complex symptomologies and varying severities. We also propose a broad clinical framework that may not only improve the current diagnostic rates, but also facilitate a personalized approach to riboflavin therapy that is low cost, safe and lead to transformative outcomes in many patients.

**Supplementary Information:**

The online version contains supplementary material available at 10.1186/s12986-023-00764-x.

## Introduction

Riboflavin or vitamin B2 is a water-soluble vitamin and an essential component of the human diet. The main food sources of riboflavin are meat and dairy as well as green vegetables. In some populations, a major contribution of riboflavin to the diet comes from fortified cereals and bread as well as nutritional supplements. Another source of riboflavin is the gut microbiome where specific intestinal resident bacteria have been demonstrated to synthesise vitamin B2 [1]. However, the contributions of the gut microbiome are generally considered to be insufficient to meet dietary needs [2, 3].

Dietary riboflavin is taken up in the human gastrointestinal tract by specialised transport protein systems localised in the brush border of the apical membrane of polarized gut enterocytes [4]. The solute carrier family members SLC52A1, SLC52A2 and SLC52A3 are primarily involved in the uptake of riboflavin with each having a different tissue-specific expression profile, sub-cellular localisation and functional properties.

**Fig. 1:**
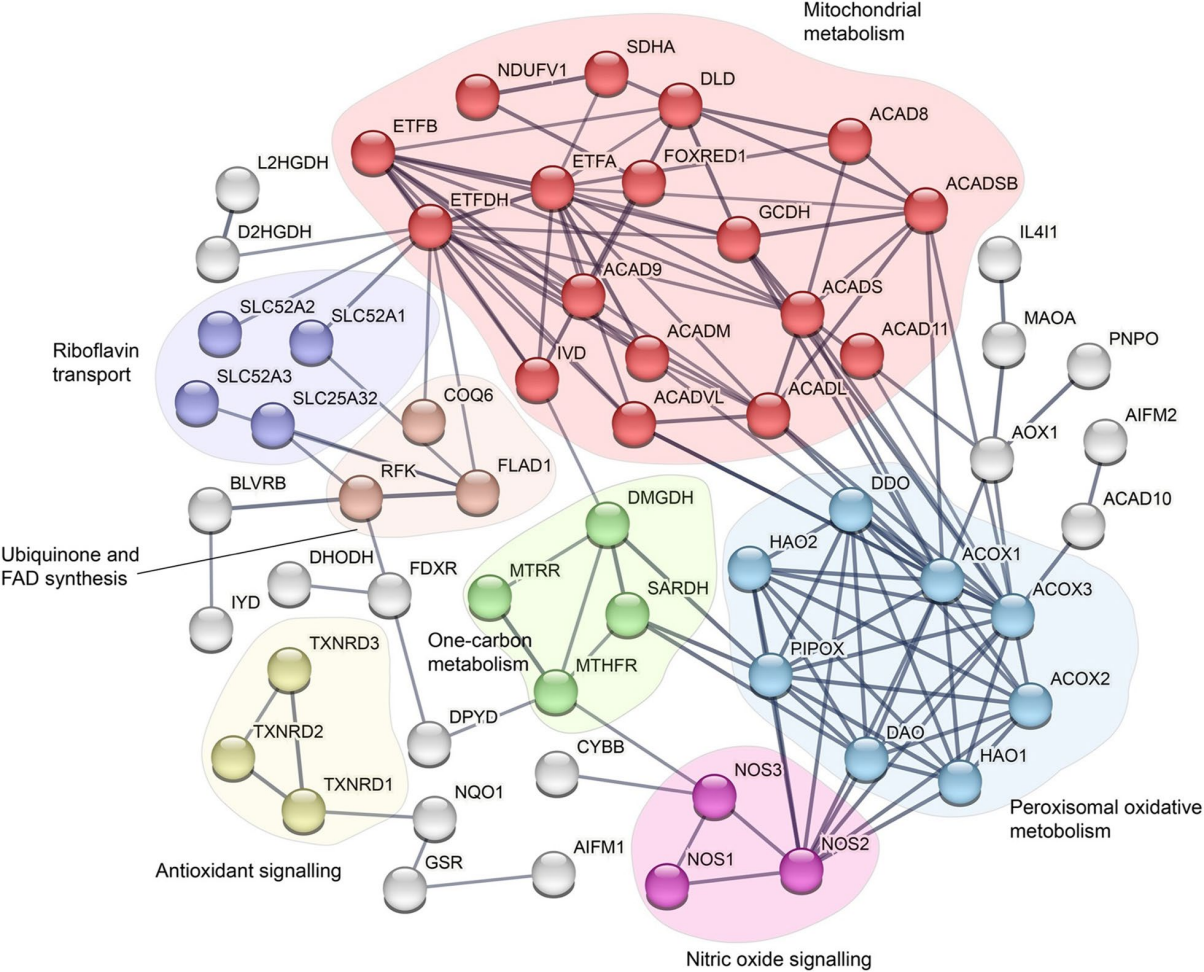
Network analysis of the human flavoproteome. Human flavoproteome genes (Additional file 1: Table S1) were subjected to network analysis using STRING (v11.5, 17/3/2023) using a high confidence interaction score of 0.7. Gene clusters were identified in which the colour nodes represent functional groups in which the thickness of the edges corresponds to confidence of association between genes. The major functional groups identified in the human flavoproteome are (i) mitochondrial metabolism (red), (ii) riboflavin transport (purple), (iii) ubiquinone and FAD synthesis (orange), (iv) antioxidant signalling (yellow), (v) one-carbon metabolism (green), (vi) nitric oxide signalling (pink) and (vii) peroxisome oxidative metabolism (blue)

Riboflavin is the precursor of the flavins, flavin mono- nucleotide (FMN) and flavin adenine dinucleotide (FAD) which play essential roles as cofactors of enzymes in diverse biochemical reactions. Flavoenzymes that utilize either FMN or FAD as cofactors commonly participate in redox reactions where the flavin tricyclic isoalloxazine ring promotes the exchange of electrons [5, 6]. Of the over 90 genes encoding flavoenzymes, 75 require FAD and 9 require FMN for their function, while the remaining 6 proteins acting as either riboflavin membrane transporters or cytosolic enzymes (Additional file 1: Table S1) [5]. Flavoproteome functional networks play key roles in (i) mitochondrial metabolism, (ii) riboflavin transport, (iii) ubiquinone and FAD synthesis, (iv) antioxidant signalling, (v) one-carbon metabolism, (vi) nitric oxide signalling and vii) peroxisome oxidative metabolism (Fig. 1).

Reflecting these diverse functional networks, genetic defects in flavoprotein genes can lead to a range of pathologies characterised by clinically broad presentations that may lead to complex dysfunctions involving defective fatty acid oxidation, abnormal acylcarnitine metabolism, neuromuscular disorders, foetal developmental defects, cardiovascular disease, metabolic acidosis and hypoketotic hypoglycaemia. In many cases, their clinical presentation occurs within the first year of life (early-onset) and is associated with high mortality rates [6–8]. However, what is particularly striking is that there is a subset of later-onset pathologies involving genetic variants of flavoprotein genes that occur within teenage years or adulthood (referred to as late-onset or adult-onset in this review) many of which are highly treatable using high-dose riboflavin therapy.

Notably, single nucleotide polymorphisms (SNPs) associated with genes encoding flavoproteins within mitochondrial, riboflavin transport and one-carbon metabolism networks have been identified as being associated with late-onset riboflavin-responsive pathologies. In this review, we will discuss the data showing how SNPs in flavoprotein genes modify individual dietary riboflavin requirements leading to late-onset metabolic pathologies and whether current evidence can lead to new treatment approaches based on a personalised nutrition approach that is informed by functional genomics. Using key examples from the networks depicted in Fig. 1, we will further discuss how specific SNPs impact on flavoprotein structure:function and propose possible mechanistic insights into their clinical aetiology.

## Riboflavin dietary requirements—role of genetics

Inadequate dietary intake of riboflavin, known as ariboflavinosis, is manifested by a range of diverse clinical presentations that can include peripheral neuropathy, fatigue, exercise intolerance, defects in lipid oxidation, anaemia and dermatological symptoms [9]. In children, ariboflavinosis presents with growth retardation while insufficient intake during pregnancy has been reported to be associated with congenital heart disease [2]. The Estimated Average Requirement (EAR) for men is 1.1 mg and for women is 0.9 mg daily (ages 19 + years) [9]. Women who are pregnant or lactating have higher EARs with recommended intakes of 1.2 mg and 1.3 mg daily respectively. Such EAR levels are based on the amount of riboflavin estimated to meet the requirements of 50% of healthy individuals [9].

While most healthy diets, that include all major food groups, are generally believed to meet riboflavin requirements, it should be noted that riboflavin together with FMN and FAD are not stored in significant amounts within the body and so regular daily intake is important in avoiding deficiencies [10]. Moreover, riboflavin requirements can vary depending on metabolic demands, pathological influence of diseases as well as the efficiency of its adsorption and uptake. Thus, while ariboflavinosis is rarely diagnosed in developed countries, subclinical deficiencies have been reported to occur in significant proportions of some populations [11, 12]. For example, it is estimated that greater than 5% of Australian adults have inadequate intake of riboflavin with the relative proportions of deficiency increasing with age (Table 1) [13, 14].

**Table 1:** Nutritional requirements of riboflavin

Age (years)	EAR (mg)		Proportion of riboflavin inadequacy (%)	
	Males	Females	Males	Females
2–3	0.4	0.4	–	–
4–8	0.5	0.5	–	–
9–13	0.8	0.8	1.0	2.9
14–18	1.1	0.9	4.6	8.4
19–30	1.1	0.9	3.4	7.7
31–50	1.1	0.9	4.4	7.8
51–70	1.1	0.9	8.7	8.1
71 and over	1.3	1.1	20.3	20.3

The table shows the proportion of population with inadequate riboflavin intake, estimate as % below Estimate Adequate Requirement (EAR) (Source Australian Bureau of Statistics [13])

While EARs and Recommended Daily Intakes (RDIs) have been an important public health measure that provide evidence-based guidelines at a population level, they may not be appropriate for specific individuals due to a range of environmental and genetic influences. In addition to dietary insufficiencies, there is a spectrum of genetic variants including SNPs that give rise to in-born errors of nutrient adsorption, transport and metabolism that can result in riboflavin requirements well above the RDI [15]. Mutations in the genes encoding flavoproteins that are associated with a range of neonatal (early-onset) metabolic pathologies and high mortality rates have been widely reported and reviewed [6–8]. However, SNPs associated with later-onset pathologies in adolescents and adults involving defective flavin metabolism are often overlooked, frequently misdiagnosed and can be associated with long-term disability if untreated [14].

## Riboflavin-responsive variants in riboflavin transport genes

Riboflavin is water soluble and is transported across the lipidic layers of biological membranes by a family of transporters [6]. Each of the riboflavin transporters has a specific tissue distribution with SLC52A1 expressed mostly in the intestinal epithelium, the placenta and skin. SLC52A2 is most highly expressed in the nervous system but is also expressed ubiquitously at lower levels in other tissues (GTEx). SLC52A3 is primarily expressed in the testis and prostate but is also found in the intestine (GTEx). Lastly, the mitochondrial transporter SLC25A32 is expressed ubiquitously with the highest expression in the bladder, adrenal gland, arteries, breast and adipose tissues (GTEx).

The pathological consequences of a specific genetic variant in an individual riboflavin transporter in terms of organ function will not only depend on its expression pattern, but also on whether the redundant expression of family members can compensate for the loss of transport functions [16]. It is for this reason that gene variants that result in impaired function of a single riboflavin transporter function do not phenocopy typical ariboflavinosis symptoms.

Notably, in otherwise healthy females who become pregnant, transient neonatal riboflavin deficiency has been shown to be associated with a rare genetic variant of SLC52A1 (*rs141935493*) in the mother rather than the foetus [17]. In such cases it has been proposed that the increased riboflavin requirements during pregnancy together with the non-redundant role of placental SLC52A1 in supplying riboflavin to the foetus result in an adult-onset deficiency leading to the inability of the placenta to adequately supply the developing foetus with riboflavin (OMIM 615026) [16–18]. Such a proposal would be consistent with SLC52A1 performing non-redundant roles in riboflavin transport in the placenta. Given that blood concentrations of riboflavin are commonly decreased during pregnancy [19], pregnant individuals with SLC52A1 mutations may require higher riboflavin intake to avoid gestational riboflavin deficiency. For example, such individuals have been shown to respond to riboflavin therapy (10-80mg/kg/day) during pregnancy to compensate for their defective SLC52A1 and to ensure adequate supply tor the foetus [16].

In addition to SLC52A1, mutations in either SLC52A2 or SLC52A3 have been reported to lead to riboflavin transporter deficiencies (RTDs) that include Brown–Vialetto–Van Laere (BVVL) syndrome (OMIM: 211530) and Fazio–Londe syndrome (FLS, OMIM: 211500). In both conditions, onset is accompanied by a blood profile of acyl-carnitines similar to that observed in Multiple Acyl-CoA Dehydrogenase Deficiency (MADD, see below) patients, suggesting a defect in the mitochondrial metabolism [20]. However in the case of BVVL, while symptoms can vary in severity, they are also characterized by progressive neuropathy, vision and hearing impairment, muscle weakness and respiratory difficulties [21]. Although the age of onset ranges from birth to the fourth decade, symptom onset most commonly occurs in the second decade of life [22]. Importantly, multiple BVVL patients with either SLC52A2 or SLC52A3 variants have been reported in which riboflavin therapy ameliorates symptoms, including later-onset disease in adults. While the therapeutic doses of riboflavin used vary widely, significant clinical responses have been observed in adults using up to 1500mg/day with no reported adverse reactions [23, 24].

Because of their heterogeneous clinical presentations, individuals with late-onset RTD pathologies involving SLC52A2 and SLC52A3 can be difficult to diagnose. In a retrospective analysis of RTD patients, more than half were misdiagnosed with amyotrophic lateral sclerosis (ALS) or distal hereditary motor neuropathy [14, 24]. In some cases, specific subclinical RTD symptoms that can be informative are only detected after extensive clinical workup [17]. For example, an 18-year-old woman with a heterozygous SLC52A3 mutation presented with severe respiratory distress requiring ventilation, extreme muscle weakness and symptoms mimicking ALS [22]. The patient responded to high dose of riboflavin (15 mg/kg/day) and after 1 year of supplementation she was walking and independent for daily activities. Given the serious and potentially irreversible progression of BVVL together with the known safety and effectiveness of riboflavin therapy in some patients, it has been recommended that the suspicion of RTD should lead to the immediate commencement of riboflavin treatment without waiting for genetic confirmation [25, 26].

The molecular mechanisms underpinning the adult-onset BVVL and FLS and their response to riboflavin remain unclear. It could be that the mutations identified in SLC52A2 or SLC52A3 result in a partial decrease in transporter activity such that higher concentrations of riboflavin are required to restore adequate uptake. However, in silico and functional analysis of the SLC52A2 and SLC52A3 pathogenic mutations predicts that they would most likely result in a complete loss of function [23, 24, 27]. While the detailed structures of SLC52A2 and SLC52A3 remain to be determined, the suggested mechanistic basis for BVVL is more likely to be due to a complete *loss-of-function* of either SLC52A2 or SLC52A3 that can be compensated through supplying high concentrations of riboflavin in order to increase the efficiency of the remaining wild-type riboflavin transporters and so restore intracellular flavin homeostasis [14, 16].

It is also not clear why some individuals with mutations in their riboflavin transporters can be seemingly healthy, sometimes for several decades, prior to the onset of disease. In cases where there is a loss of function in one riboflavin transporter, it might be that as the requirement for dietary riboflavin increases with age, it eventually exceeds the ability of the remaining functional transporters to deliver sufficient riboflavin in some tissues. It could also be that any decrease in riboflavin intake due to a deterioration in diet quality could be an important contributor. For example, in a case study of a 35-year-old woman, the onset of BVVL symptoms was found to be associated with deterioration of the quality of the patient’s diet including decreased riboflavin intake. The patient presented with weight loss, severe muscle weakness and progressive dysphagia, dysphonia and dyspnoea. These symptoms had started about 8 weeks before hospital admission and had rapidly worsened. DNA sequencing identified Val413Ala (*rs2676066871*) and Asp461Tyr (*rs2676066871*) compound heterozygous mutations in the SLC52A3 gene. Both the mutations were predicted to be partially disruptive for SLC52A3 transporter function, thereby leaving the patient susceptible to the decreased dietary riboflavin. The treatment of this patient with 200mg/day riboflavin dramatically resolved symptoms [28].

### Mitochondrial riboflavin transport

Another important regulator of riboflavin metabolism is the mitochondrial transporter encoded by the SLC25A32 gene. This multi-transmembrane protein belongs to a family of mitochondrial carriers and has been reported to transport FAD across the inner mitochondrial membrane. Mutations in SLC25A32 result in reduced FAD availability for mitochondrial flavoenzymes including those involved in fatty acid β-oxidation and respiratory-chain oxidative phosphorylation as well as 1C metabolism (Fig. 1) [29, 30]. For example, Schiff and colleagues reported the case of a 14-year-old girl presenting with a fatty acid oxidation dysfunction and intermittent exercise intolerance in which symptoms were substantially improved after riboflavin treatment (dose not reported). Exon sequencing identified Tyr142Ter (*rs147014855*) and Arg147His (*rs142329098)* compound heterozygous mutations in SLC25A32 [31]. In a separate study, a homozygous Gly91Val (rs142329098) variant was identified in three patients who responded to riboflavin therapy (30–100 mg/day) with significant improvement in clinical symptoms [32].

In the absence of detailed structural analysis of mammalian riboflavin transporter proteins, the structure:function implications of specific mutations are difficult to determine. While it is proposed that the riboflavin transporters are multi-pass transmembrane proteins, there is a lack of consensus in terms of the modelling and experimental data regarding the number of transmembrane domains, the mechanism of riboflavin transport and possible quaternary structures [4, 33]. Thus, further work will be required to determine whether there might be hot-spots within riboflavin transporters in which mutations may lead to late-onset riboflavin-responsive pathologies.

## Riboflavin-responsive variants in enzymes involved in FAD and FMN synthesis

Dietary riboflavin is converted into its active forms FMN and FAD by two enzymatic activities. Firstly, riboflavin kinase (RFK) catalyses the phosphorylation of riboflavin to FMN. The second biosynthetic reaction is an adenylation that transforms FMN to FAD and is catalysed by FAD synthetase (FADS) encoded by the Flavin Adenine Dinucleotide Synthetase 1 (FLAD1) gene. Human FADS exists in at least 2 different isoforms (FADS1 and FADS2), both of which contain a C-terminal catalytic domain which is sufficient to catalyse FAD synthesis, and a N-terminal molybdopterin binding (MPTb) domain [34]. The longer FADS1 isoform has a mitochondrial targeting sequence while the shorter FADS2 form results from alternative splicing and is localized in the cytosol [35, 36]. While FADS enzyme activity is responsible for supplying the flavoproteome with sufficient FAD, it also functions as a FAD-binding protein [37].

To date, no RFK variants associated with riboflavin-responsive pathologies have been identified, however, a complete RFK deficiency in mice has been reported to result in embryonic lethality [38]. On the other hand, multiple FLAD1 gene variants have been identified with disease associations (OMIM #610,595). For example, multiple distinct gene variations in FLAD1 affecting 7 families have been reported that demonstrate a wide range of clinical severity and responsiveness to riboflavin [36]. In some individuals, biallelic Ser495del variants were associated with riboflavin-responsive MADD-like illness. In other patients, compound heterozygotes consisting of an Arg530Cys (*rs771466122*) and an allele composed of a frameshift mutation, either Val191Glnfs (*rs876661310)* or Phe279Serfs (rs876661311) in the N-terminal MPTb domain were associated with adult-onset riboflavin-responsive disease [36]. In a separate study, adult-onset muscle weakness and MADD-like symptoms that were exacerbated by either fasting or pregnancy were also identified in a compound heterozygote patient consisting of a frameshift mutation in the MPTb domain and a Arg530Cys mutation. Importantly, patients with compound heterozygotes composed of the Arg530Cys and N-terminal frameshift mutations demonstrated a significant improvement in symptoms following treatment with 100mg/day riboflavin [39].

**Fig. 2:**
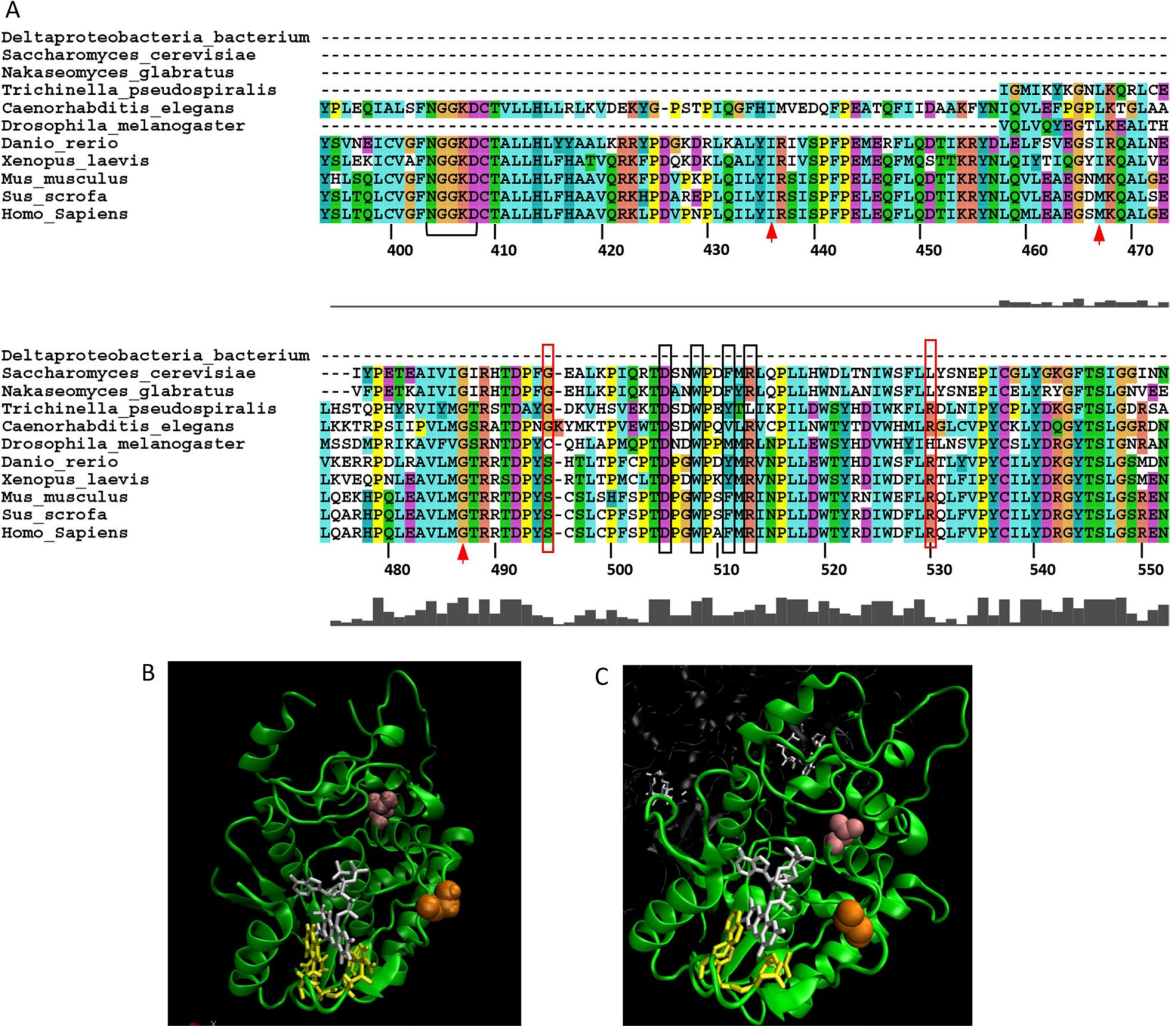
Riboflavin-responsive mutants of FADS. **A** The alignment for the FADS catalytic domain was generated for known (or predicted) FADS sequences from human (*Homo sapiens* NP_079483.3), pig (*Sus scrofa*, XP_001929410.3), mouse (*Mus musculus*, NP_796015.2), frog (*Xenopus laevis*, XP_041427932.1), zebra fish (*Danio rerio*, NP_001003997.1), worm (*Caenorhabditis elegans*, NP_001022287.1), nematode (*Trichinella pseudospiralis* KRX89489.1), fruit fly (*Drosophila melanogaster*, NP_727648.1), yeast (*Nakaseomyces glabratus*, KTB21675.1), yeast (*Saccharomyces cerevisiae* EIW11165.1) and bacteria (*Proteobacteria*, MBQ30972.1) using ClustalX [113]. Amino acid numbers for the human sequence are indicated. Red boxes indicate the locations of the Ser495del and Arg530Cys human variants associated with adult-onset riboflavin-responsive disease. The bracket indicates the NGGKD motif that is important for FAD binding. Red arrows indicate Ile436, Met467 and Gly487 that are known to be important for FAD binding. Black boxes indicate Asp505, Trp508, Phe511 and Arg513 that are predicted to interact with the isoalloxazine rings. Ribbon diagrams (generated in VMD 1.9.1 [114] of the crystal structures of *Saccharomyces cerevisiae Fad1p* **B** [40] and *Candida glabrata* FMNAT **C** [41] in complex with FAD (white) are shown. Black boxed amino acids from panel A that are known to interact with isoalloxazine rings of FAD are highlighted in yellow (**B**, **C**). The conserved glycine (orange) in Fad1p and FMNAT that lies at the equivalent position to Ser495 in human FADS together with the conserved leucine (pink) at the equivalent position to Arg530 in human FADS flank the isoalloxazine ring-binding amino acids (yellow) in the primary structure

While the crystal structure of human FADS has not been determined, homology modelling based on the crystal structures of fungal homologues in *Saccharomyces cerevisiae* (Fad1p) and *Candida glabrata* (FMNAT) has allowed important insights into FAD binding [40–42]. While Fad1p and FMNAT do not contain a MPTb domain, their crystal structures are highly superimposable and are > 49% conserved with the amino acid sequence of human FADS. Structural modelling of FADS suggests that FAD adopts a bent conformation within the catalytic domain in which there are extensive interactions with the tricyclic isoalloxazine and adenine moieties [34, 40, 42]. Based on this modelling, the conserved 404-NGGKD-408 motif (Fig. 2A, black bracket) in human FADS together with the conserved Ile436, Met467 and Gly487 (Fig. 2A, red arrows) form a pocket that is critical for binding the adenine rings of FAD. Importantly, each of these amino acids lies at N-terminal of the riboflavin-responsive Ser495del truncation mutant which retains FAD binding capacity, albeit at lower amounts. Thus, the 404-NGGKD-408 motif together with Ile436, Met467, Gly487 are likely to contribute to the docking site for the adenine moiety of FAD binding. In addition, Asp505, Trp508, Phe511 and Arg513 of human FADS are highly conserved amino acids among eukaryotes (Fig. 2A, black boxes) and are predicted to interact with the tricyclic isoalloxazine rings of FAD [34, 40, 42]. As shown in Fig. 2, the amino acids corresponding to Asp505, Trp508, Phe511 and Arg513 in Saccharomyces cerevisiae Fad1p (Fig. 2B, yellow amino acids) and Candida glabrata FMNAT (Fig. 2C, yellow amino acids) form a binding pocket for the tricyclic isoalloxazine rings of FAD. Thus, the loss of Asp505, Trp508, Phe511 and Arg513 due to the upstream Ser495del truncation mutant would likely have impacts on either FAD binding or affinity leading to FADS protein instability particularly under conditions where riboflavin concentrations are reduced [34, 40, 42, 43]. Consistent with this notion, the Ser495del mutant not only exhibits reduced binding to FAD compared to the wild-type protein, but also has a higher in vitro sensitivity to proteolytic degradation which can be rescued by the addition of a molar excess of FAD [36]. Such observations would be consistent with clinical reports in which individuals with Ser495del (*rs876661309*) variants demonstrate riboflavin-responsive pathologies [36].

The structure:function implications of other variants of FADS in terms of riboflavin-responsive phenotypes is less clear. For example, late-onset riboflavin-responsive patients with one allele expressing the Arg530Cys mutant (Fig. 2A) have a second allele that encodes a frameshift mutation within the N-terminal MPTb domain that would be predicted to be a loss-of-function mutant [36, 39]. More recently the case of an 8-year-old boy was reported with homozygosity for a truncation variant of FLAD1 (rs199979286), correlating with a reduced presence of the corresponding protein as well as FAD synthesis. This patient showed a clinical response to riboflavin treatment [44]. However, it has been shown that at least in some MPTb frameshift mutations, the use of downstream in-frame ATG codons allows the expression of a cytoplasmic FADS domain that may permit the expression of an active catalytic domain. While the functional consequences of the Arg530Cys mutant are also difficult to predict, analysis of the crystal structures of *Saccharomyces cerevisiae* Fad1p (Fig. 2B, pink amino acid) and *Candida glabrata* FMNAT (Fig. 2C, pink amino acid) would suggest that the leucine in the equivalent position to Arg530 of the human FADS is not directly involved in FAD binding. Such a conclusion has also been suggested by other investigators following in silico modelling of the human FADS based on the Fad1p and FMNAT crystal structures [34, 40, 42]. Although the Arg530Cys protein appears to be less stable than the wild-type protein, the instability is not rescued by an excess of FAD suggesting that the labile nature of the protein is not due to decreased FAD binding. Thus, the structural basis for the riboflavin-responsiveness of individuals expressing the Arg530Cys may require additional investigations.

## Riboflavin-responsive variants affecting mitochondrial metabolism

### Electron transfer flavoprotein dehydrogenase (ETF-QO)

Genetic disorders that involve in-born errors of flavoprotein metabolism that are characterized by early-onset multiple acyl-CoA dehydrogenase deficiency (MADD; OMIM #231680) have long been recognized principally for their severe neonatal phenotypes and high mortality rates. In particular, early-onset MADD can arise from loss-of-function variants in the electron transfer flavoprotein A (ETFA), electron transfer flavoprotein B (ETFB) or the electron transfer flavoprotein dehydrogenase (ETFDH) genes. The expression of such variants results in defects in the transfer of electrons from acyl-CoA dehydrogenase to the respiratory chain leading to impaired mitochondrial beta oxidation and ATP production [45].

While the autosomal recessive mutations in ETFA, ETFB and ETFDH that give rise to such neonatal-onset MADD have been widely described [46], late-onset MADD has received less attention. Affected individuals can develop a spectrum of symptoms in adolescence or adulthood that can include exercise intolerance, myalgia, chronic fatigue, metabolic acidosis, cognitive impairment and post-exercise pain [47]. Diagnosis of late-onset MADD can be challenging and requires the investigation of multiple biochemical biomarkers that may include urine organic acids as well as blood acyl-carnitines and lactate.

**Fig. 3:**
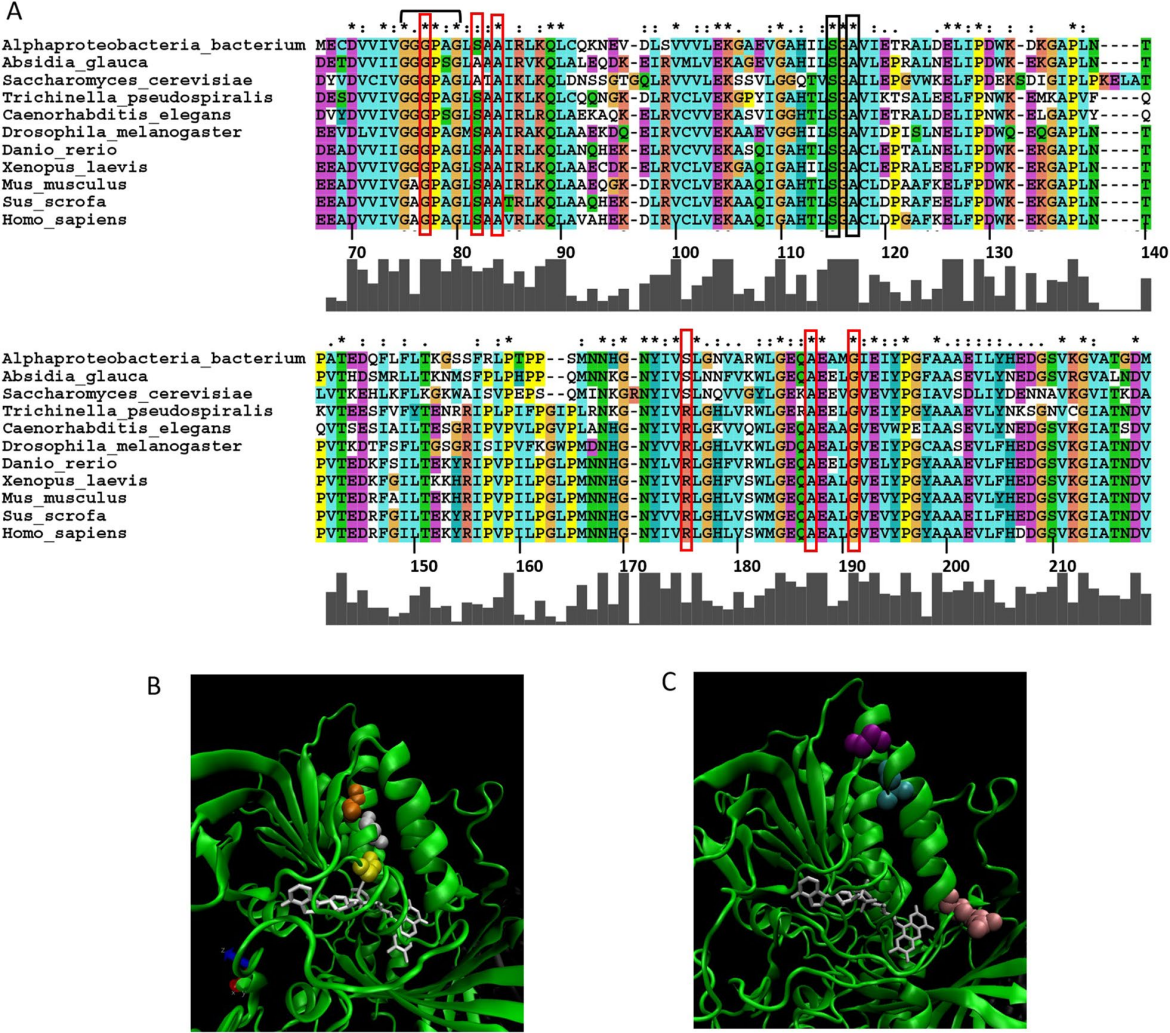
Riboflavin-responsive mutants of ETF-QO. The alignment was generated for known (or predicted) ETF-QO sequences from human (*Homo sapiens* AAB24227.1), pig (*Sus scrofa*, XP_003129051.1), mouse (*Mus musculus*, NP_080070.2), frog (*Xenopus laevis*, NP_001087869.1), zebra fish (*Danio rerio*, NP_001004598.1), worm (*Caenorhabditis elegans*, NP_001379625.1), nematode (*Trichinella pseudospiralis* XP_003377314.1), fruit fly (*Drosophila melanogaster*, NP_001246231.1), yeast (*Saccharomyces cerevisiae* Q08822), fungus (Absidia glauca A0A168RVN9) and bacteria (*Proteobacteria*, MCR9071071.1) using ClustalX [113]. Amino acid numbers for the human sequence are indicated. Red boxes indicate the locations of the human variant amino acids Gly77, Ser82, Ala84, Arg175, Ala187 and Gly191 that are associated with adult-onset riboflavin-responsive disease. The GXGXXG adenine binding motif is indicated with a black bracket. Black boxes indicate Ser115 and Ala117 that are involved in hydrogen bonding with the tricyclic isoalloxazine rings of FAD. Two different views of ribbon diagrams (generated in VMD 1.9.1) [114] of the crystal structure of porcine ETF-QO **B**, **C** [49] in complex with FAD (white) are shown. **B** Gly77 (yellow), Ser82 (grey) and Ala84 (orange) that are highlighted in the red boxes of panel A are subject to mutations that are associated with riboflavin responsive disease. **C** Arg175 (pink), Ala187 (cyan) and Gly191 (purple) that are highlighted in the red boxes of panel **A** are subject to mutations that are associated with riboflavin responsive diseases

While rarely fatal, late-onset MADD can be associated with decades of disability and poor quality-of-life measures. Importantly, a wide variety of gene ETFDH variants, that encode the flavo-enzyme ETF-ubiquinone oxidoreductase (ETF-QO), have been identified in individuals with late-onset MADD symptoms, many of which can be substantially improved following riboflavin therapy (Table 2). For example, an Ala84Thr (*rs121964954*) variant of ETF-QO has been associated with late-onset MADD in Southern China where the carrier frequency is estimated to be 1.35% (Fig. 3A, red box). Patients present with progressive and extreme muscle weakness, exercise intolerance and acyl-carnitine blood profiles typical of MADD. In most patients, riboflavin therapy (30-195 mg/day) provides substantial improvements to symptoms (Table 2) [48].

**Table 2:** Genes variants associated with late-onset riboflavin-responsive phenotypes

Gene name/Id	Protein	Gene Ontogeny Classification/tissue gene expression	Physiological functions	Clinical Features associated with genetic variants responding to riboflavin supplementation	Variants involved in late onset and riboflavin responsive symptoms	OMIM Phenotype	Riboflavin supplementation	References
*Riboflavin transporters*								
SLC52A1/55065	hRFT1	Membrane transporter/ Placenta and intestine	Riboflavin membrane transport	Transient neonatal riboflavin deficiency/maternal deficiency Transient MADD Phenotype	*rs346822, rs2304445,* *rs141935493*	615,026	50 or 100 mg/day	[7, 18]
SLC52A2/79581	hRFT3	Membrane transporter/ brain and most other tissues	Riboflavin membrane transport	Riboflavin deficency Brown-Vialetto-Van Laere Syndrome (BVVLS)	*rs117500243,* *rs397514538*, *rs377740960,*	614,707	50 mg/kg/day in paediatric patients and 1500 mg/day in adult patients	[7, 24, 27, 46, 106–108]
SLC52A3/113278	hRFT2	Membrane transporter/ testis/ intestine	Riboflavin membrane transport	Riboflavin deficency Brown-Vialetto-Van Laere Syndrome (BVVLS) Fazio-Londe syndrome Late onset	*rs267606, rs140360713, rs1439187603, rs1431398048*	211,530	10–25 mg⁄kg⁄day or 200 mg/day	[22, 28, 109]
SLC25A32/81034	MFTC	Ubiquitous	FAD and folate mitochondrial transport	Recurrent exercise intolerance	rs147014855 rs142329098 rs1249586962	616,839	10-30mg/day	[31]
*FAD synthesis*								
FLAD1/80308	FAD synthase	Converting FMN into FAD/ Ubiquitous expression	FAD biosynthesis	lipid storage myopathy and multiple-respiratory-chain deficiency elevation of multiple acylcarnitines, increased urinary organic acids	*rs876661310, rs876661311, rs771466122, rs876661309*	255,100	100mg/day	[36]
*Flavin dependent enzymes*								
ETFDH/2110 ETFA/2108 ETFB/2109	Electron transfer flavoprotein-ubiquinone oxidoreductase	Accepts electrons from ETF and reduces ubiquinone	Transport of electrons mitochondrial respiratory chain	MADD (type III) Exercise intolerance, myalgia, chronic fatigue, metabolic acidosis	*rs121964954, rs887871605, rs762928354, rs887871, rs121964955, rs369912835, rs147219158, rs760234838, rs780768015*	231,680	100 -300mg/day	[48, 50, 51, 110, 111]
MTHFR/4524	Methylenetetrahydrofolate reductase	Reductase in one-carbon-metabolism/ ubiquitous	Converting 5,10-methylenetetrahydrofolate to 5-methyltetrahydrofolate	Associated with increased incident hypertension and increased circulating homocysteine	*rs1801133*	236,250	16 mg/day	[80, 82, 83, 112]
ACAD9/28976	Acyl-CoA dehydrogenase 9	Mitochondrial/ubiquitously expressed	Assembly of the mitochondrial CI, Fatty Acid Beta-Oxidation	Growth retardation, Exercise intolerance	*rs762521317 rs1057518752* *rs779610933, rs863224844,* *rs149753643, rs777282696, rs1553732136*	611,126	40mg/day	[61, 62]

Genes involved in riboflavin metabolism requiring higher intakes of the vitamin, can be divided in three groups – (i) Riboflavin transporters, (ii) FAD synthesis, (iii) Flavin dependent enzymes

Analysis of the crystal structure of porcine ETF-QO provides insights into the potential structure:function consequences of the Ala84Thr variant and why it might be associated with a riboflavin-responsive phenotype. The Ala84 amino acid of the porcine ETF-QO lies immediately downstream of a GXGXXG adenine binding motif in the alpha 1 (α1) helix (Fig. 3A, bracket) [49]. Within this context, the α1 helix of ETF-QO appears to represent a hot-spot for adult-onset riboflavin-responsive MADD. For example, nearby variants Ser82Phe (*rs887871605)* and Gly77Ser (not in dbSNP) have also been reported to be associated with adult-onset riboflavin-responsive MADD (Table 2) [50, 51]. As shown in Fig. 3B, Gly77 (yellow amino acid), Ser82 (grey amino acid) and Ala84 (orange amino acid) lie within the α1 helix of ETF-QO that forms part of the binding pocket for the pyrophosphate group of FAD and so would be predicted to be important for flavin binding. It is also notable that each of these variant amino acids is highly conserved from bacteria to humans suggesting evolutionary selection for FAD binding and enzymatic activity (Fig. 3A, red boxes).

In addition, there also appears to be a cluster of variants in the α4 helix of ETF-QO that are associated with riboflavin-responsive disease. For example, the Arg175His (*rs121964955*), Ala187Val (*rs369912835*) and Gly191Ser (*rs147219158*) variants (Fig. 3A, red boxes) all lie within the α4 helix and have also been associated with late-onset riboflavin-responsive MADD [50–53]. As shown in Fig. 3C, Arg175 (pink amino acid), Ala187 (cyan amino acid) and Gly191 (purple amino acid) all lie in the α4 helix that forms part of the binding pocket for FAD. Significantly, the N-terminus of the α4 helix lies adjacent to Ser115 and Ala117 (Fig. 3A, black boxes) that are important for hydrogen bonding with the tricyclic isoalloxazine rings of FAD (Fig. 3, black boxes) [49]. Thus, in addition to α1, the α4 helix also appears to be a hotspot for late-onset riboflavin-responsive MADD and is likely to be consistent with the high conservation of Arg175, Ala187 and Gly191 across multiple species (Fig. 3A, red boxes).

One mechanism that may underly the clinical responsiveness of patients harbouring such ETF-QO mutations could be that high dose increasing riboflavin therapy leading to high intracellular levels of FAD could stabilize the active enzyme conformation. For example, Cornelius *et. al.* have shown in cell culture models in which riboflavin-responsive ETF-QO variants were ectopically expressed that high FAD levels can promote either protein folding or stability consistent with the proposal that FAD may act as a chemical chaperone. [54] In line with these findings, multiple riboflavin-responsive ETF-QO variants were also shown to be more prone to denaturation at higher temperatures (42 °C) [54].

Consistent with the proposal that FAD may function as a chemical chaperone, protein levels of the Ala84Thr and Arg175His mutants were reduced and their half-lives were significantly shorter in primary samples isolated from late-onset MADD patients [55]. Furthermore, a variant of the related family member, ETFβ-Asp128Asn, demonstrated reduced conformational stability in primary patient samples and increased susceptibility to proteolytic degradation which could be rescued by high concentrations of FAD when compared to the wild-type ETFβ [45, 56]. Thus, under either physiological or pathological conditions where intracellular FAD concentrations are reduced, ETF-QO (or ETFβ) variants with reduced affinity for FAD may become unstable and so reducing overall mitochondrial beta oxidation and ATP production.

For some ETF-QO mutations associated with riboflavin-responsive disease, the structure:function impacts on FAD binding remain uncertain. For example, the Gly429Arg *(rs759044284*), Val444Ala (*rs760234838*) and Asp511Asn (*rs780768015*) mutations have been reported to be associated with late-onset riboflavin-responsive MADD [51, 52, 54]. However, in the crystal structure of porcine ETF-QO, both variants are located on the protein surface distant from the FAD binding pocket (not shown). In such cases, it could be that these variants promote long-distance conformational changes that reduce FAD binding or affinity leading to protein instability and degradation.

#### Acyl-CoA dehydrogenase 9 (ACAD9)

Acyl-CoA dehydrogenase 9 (ACAD9) is a FAD-dependent acyl-CoA dehydrogenase involved in mitochondrial metabolism (Fig. 1) [57]. While there is evidence that ACAD9 can catalyse dehydrogenase reactions involved in fatty acid beta-oxidation and amino acid catabolism, both genetic and biochemical studies point toward primary roles in the assembly of mitochondrial complex I (CI) and the generation of ATP by oxidative phosphorylation [58].

A range of pathogenic gene variants of ACAD9 have been identified that can lead to a collection of neonatal phenotypes that include metabolic acidosis, Leigh syndrome (mitochondrial encephalopathy) and hypertrophic cardiomyopathy. As with other pathologies involving FAD-dependent enzymes (e.g. ETF-QO and FADS, see above), early-onset disease associated with ACAD9 gene variants is associated with poor prognosis. For example, in a large multi-centre study of 70 patients with diverse ACAD9 variants, Repp et al*.* reported a > 50% fatality rate within the first year of life.[59].

In contrast to neonatal disease, most individuals with ACAD9 mutations in which age of symptoms presentation is more than 1 year old have been reported to be responsive to riboflavin therapy and their overall survival was > 80% [59]. For the later-onset patients, symptoms commonly include lactic acidosis, cardiomyopathy, muscular weakness, exercise intolerance and neurological atrophies associated with intellectual disability. While the specific dosage and duration of riboflavin therapy were only available for a minority of patients, such findings highlight the clinical importance of rapid diagnosis and early treatment (Table 2) [59].

**Fig. 4:**
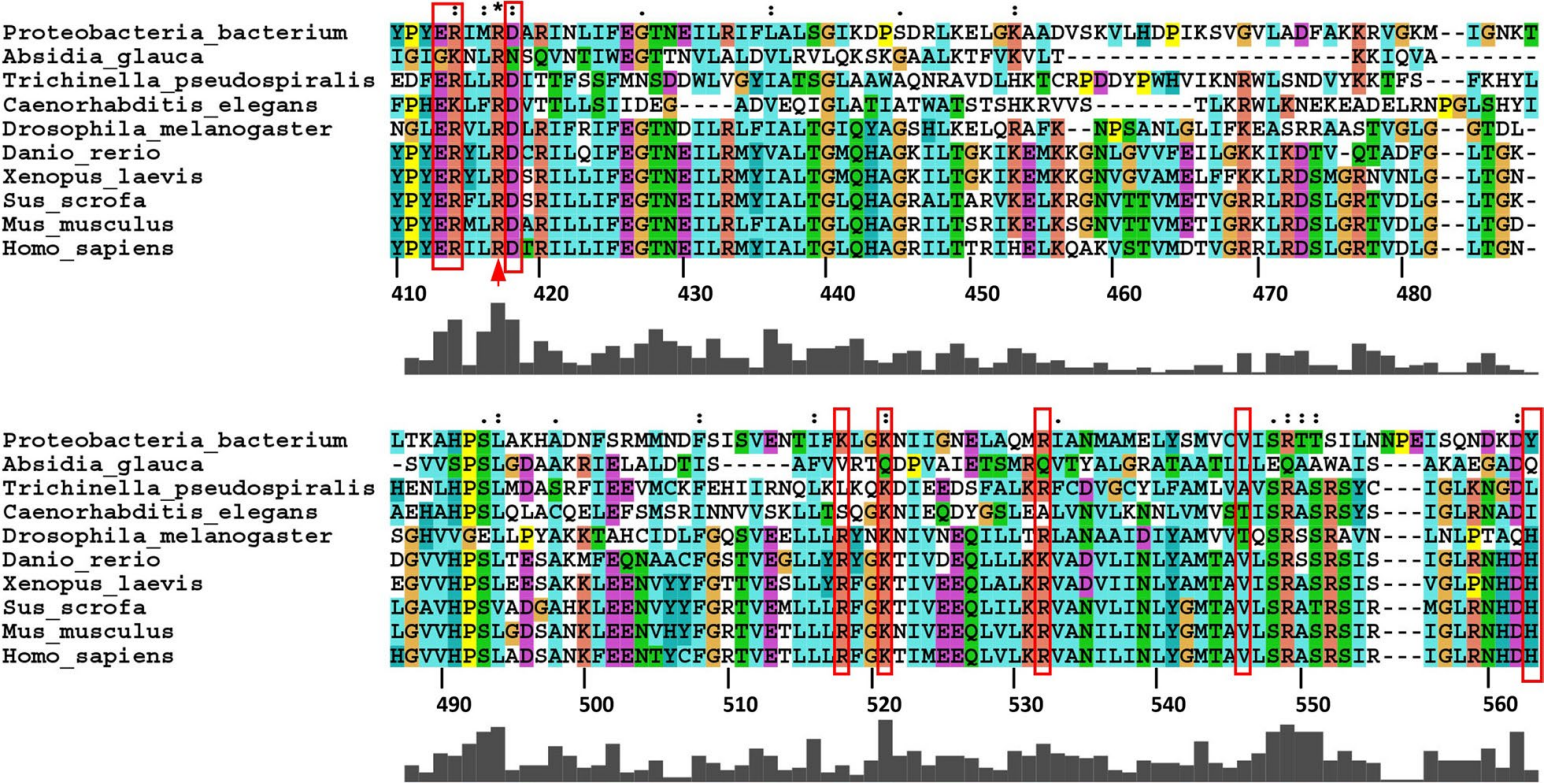
Riboflavin-responsive mutants of ACAD9. The alignment was generated for known (or predicted) ACAD9 sequences from human (*Homo sapiens* NP_054768.2), pig (*Sus scrofa*, XP_020925064.1), mouse (*Mus musculus*, NP_766266.3), frog (*Xenopus laevis*, NP_001086893.1), zebra fish (*Danio rerio*, NP_001038772.1), worm (*Caenorhabditis elegans*, NP_501452.1), nematode (*Trichinella pseudospiralis* KRX97230.1), fruit fly (*Drosophila melanogaster*, NP_001286611.1), fungus (*Absidia glauca*, ABSGL08664) and bacteria (*Proteobacteria*, MCE3013325.1) using ClustalX 2.1 [113]. Amino acid numbers for the human sequence are indicated. Red boxes indicate the locations of the human variant amino acids Glu413, Arg414, Asp418, Arg518, Lys521, Arg532, Val546 and His563 that are associated with adult-onset riboflavin-responsive disease. The red arrow indicates the location of Arg417 for which mutants would be predicted to be associated with riboflavin-responsive diseases

Gaining insight into the potential structural consequences of riboflavin-responsive ACAD9 mutants is difficult as crystal structures are not available. However, Nouws *et. al.* have performed in silico homology modelling using a related protein, human very long-chain acyl-CoA dehydrogenase (VLCAD, Accession NP_000009) to develop a predicted structure of human ACAD9 [57, 60]. The amino acid sequence of human ACAD9 (Accession NP_054768.2) is 47% identical (64% conserved) to the human very long-chain acyl-CoA dehydrogenase (VLCAD, Accession NP_000009) and both proteins function as homodimers bound to the inner mitochondrial membrane. Using this homology model, the predicted tertiary location of ACAD9 variants that are associated with riboflavin-responsive phenotypes in patients would suggest structural hotspots that could inform clinical intervention. For example, riboflavin-responsive variants of ACAD9 reported by Repp *et. al. a*nd Dewulf *et. al.* that include Arg518His (*rs781149699*), Arg532Trp (*rs377022708*), Val546Leu (*rs139352788*) and His563Asp (*rs1057518752*) (Fig. 4, red boxes) are predicted to lie within a three-helix bundle (encompassing Arg518-His563) composed of two anti-parallel helices between which lies a short spacer helix [59, 61]. Where riboflavin treatment data was available, patients demonstrated dramatic improvements in symptoms and remained clinically stable on 40-100mg/day riboflavin (Table 2) [59, 61].

An additional variant has been identified within the same predicted 3-helix bundle of ACAD9 in a case of child-onset optic and peripheral neuropathy. Genetic analysis identified a compound heterozygote in which one allele resulted in a Phe120Ser frame shift (*rs863224844*) that was likely associated with a loss of function and the second allele resulted in a Lys521Arg *(rs779610933*) missense mutation (Fig. 4, red box) [62]. After 2 months of riboflavin supplementation of 15mg/day, the patient showed improved visual function and increased lower limb muscle strength (Table 2) [62]. Based on the homology model of ACAD9, the highly conserved Lys521 is predicted to lie within the short spacer helix of the three-helix bundle that contributes to hydrogen bonds with FAD [57, 60, 63].

Together, these reports suggest that the three-helix bundle in ACAD9 that includes the variants Arg518His, Lys521Arg, Arg532Trp, Val546Leu and His563Asp represent an important hotspot (amino acids Arg518-His563) for mutations that are riboflavin-responsive (Fig. 4). While the precise mechanisms by which the variants in this region lead to riboflavin-responsive disease remains unclear, it is possible that they induce conformational changes leading to reduced FAD binding affinity that, at least partially, can be overcome by increasing FAD concentrations through riboflavin therapy.

There is an additional cluster of ACAD9 variants that include Glu413Lys (*rs149753643*), Arg414Cys (*rs777282696*) and Asp418Gly (*rs1553732136*) that have also been reported to be associated with riboflavin-responsive pathologies (Table 2) (Fig. 4, red boxes) [59]. In the homology model of Nouws et. al., these amino acids are predicted to lie within an alpha helix that is immediately N-terminal to a short, highly conserved, inter-helical loop that forms multiple hydrogen bonds with the isoalloxazine rings and ribitol chain of FAD [57, 60, 63]. Thus, the hotspot encompassed by Glu413-Asp418 of ACAD9 is predicted to have important roles in mediating FAD binding (Fig. 4, red boxes).

Given the high mortality rates for individuals with ACAD9 mutations, the importance of the two ACAD9 riboflavin-responsive hotspots identified herein that are encompassed by amino acids Glu413-Asp418 and Arg518-His563 (Fig. 4, red boxes) may allow improved clinical strategies for the identification of patients amenable to targeted therapeutic intervention. For example, in a study of individuals with CI deficiencies, two individuals with Arg417Cys variants in ACAD9 both died in childhood without a definitive diagnosis. [64] While it appears that neither individual received riboflavin treatment, the structure–function predictions outlined above would suggest that the Arg417Cys variant would lie within the Glu413-Asp418 riboflavin-responsive hotspot (Fig. 4, red arrow) for which patients would benefit from riboflavin therapy.

### Common polymorphisms in MTHFR and possible riboflavin-responsive phenotypes

Methylenetetrahydrofolate reductase (MTHFR) is the FAD-dependent rate-limiting enzyme that catalyses the conversion of 5,10-methylenetetrahydrofolate to 5-methyltetrahydrofolate and is part of the evolutionarily conserved one-carbon (1C) metabolic pathway (Fig. 1). The generation of 1C units (i.e. methyl groups) is essential for the biosynthesis of nucleic acids, amino acids, creatine, and phospholipids. Mutations in the MTHFR gene lead to inborn errors in folate metabolism syndromes with more than 100 variants with clinical significance identified to date (OMIM #607093). The common MTHFR C677T missense variant (*rs1801133*) results in an Ala222Val substitution in the MTHFR protein and has a worldwide homozygous prevalence of 10% (ranging from 4 to 18% in the United States, 12% in individuals of African ancestry, 20% in northern China to as high as 32% in Mexico and 35% in individuals of European ancestry) [65, 66].

The homozygous T-allele of the C677T polymorphism (termed 677TT hereafter) is associated with higher plasma concentrations of homocysteine and is recognised as a significant risk factor for neural tube defects [67, 68]. In addition, meta-analyses of observational studies have reported that the C667T polymorphism is associated with an increased risk of atherosclerosis, stroke and coronary heart disease although the association was not apparent in some geographical regions where folate intake is higher [67, 69].

While folate supplementation is now widely recommended during pregnancy to reduce the likelihood of neural tube defects, it remains unclear whether such supplementation in individuals harbouring the C677T variant may reduce plasma homocysteine concentrations and the risk of cardiovascular disease. In a trial examining risk of ischaemic stroke, folic acid supplementation in combination with enalapril (an angiotensin converting enzyme inhibitor) significantly reduce plasma homocysteine concentrations and the risk of ischaemic stroke. However, the responses were not significantly different after stratification of patients based on their C677T genotype [70]. Furthermore, in a meta-analysis of clinical trials, folate supplements provided no benefit in terms of decreasing blood clots, heart disease, cancer or overall mortality [71]. While further studies are required, such findings suggest that the biochemical consequences of the C677T variant that increase cardiovascular risk cannot be ameliorated by folate supplementation.

**Fig. 5:**
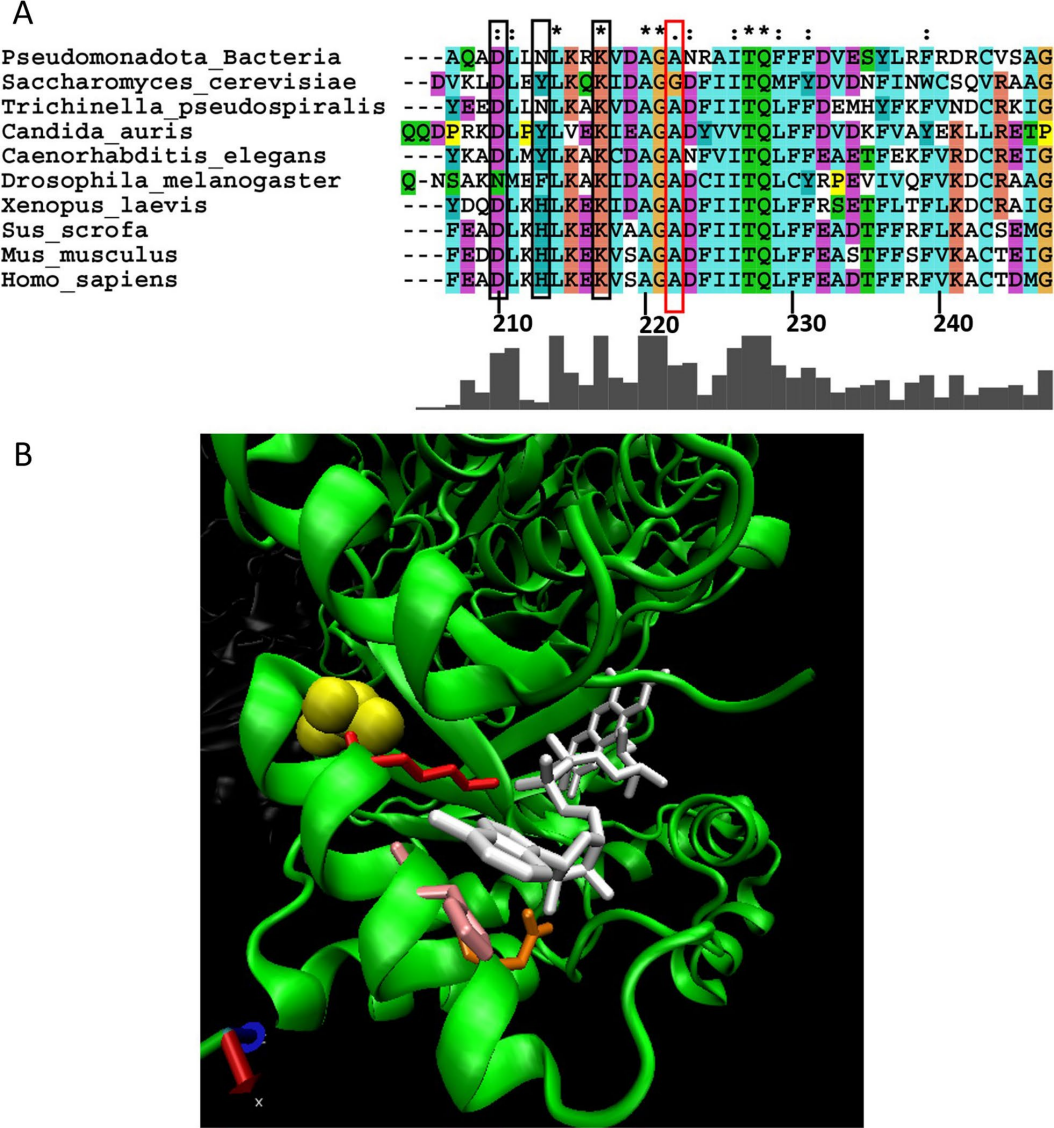
Riboflavin-responsive mutants of MTHFR. The alignment was generated for known (or predicted) MTHFR sequences from human (*Homo sapiens* NP_005948.3), pig (*Sus scrofa*, XP_005665053.1), mouse (*Mus musculus*, AAD20313.1), frog (*Xenopus laevis*, XP_018083188.1), worm (*Caenorhabditis elegans*, Q17693.2), nematode (*Trichinella pseudospiralis* KRZ29695.1), fruit fly (*Drosophila melanogaster*, NP_648462.1), fungus (*Candida auris,* GBL48066.1), yeast (*Saccharomyces cerevisiae,* KAF4004889.1) and bacteria (*Pseudomonadota, WP_000007523.1*) using ClustalX version 2.1 [113]. Amino acid numbers for the human sequence are indicated. The red box indicates the location of Ala222. **B** A ribbon diagram (generated in VMD 1.9.1) [114] of the MTHFR crystal structure in complex with FAD (white) is shown. [72] Ala222 (yellow) lies at the C-terminal end of the a6 helix. Black boxed amino acids from panel **A** that are known to interact with FAD are located in a6 helix with Asp210 (orange side chain) and His213 (pink side chain) and Lys217 (red side chain) making contacts with the adenine rings

While the precise molecular mechanisms responsible for the increased cardiovascular risk in homozygous 677TT individuals remains to be determined, it is notable from the human MTHFR crystal structure (PDB: 6FCX) that Ala222 (Fig. 5A, red box) lies at the C-terminal end of the α6 helix which forms part of the FAD binding pocket (Fig. 5B, yellow amino acid). The nearby Lys217 (Fig. 5B, red side chain) forms a hydrogen bond with the ribitol chain of FAD and both Asp210 (Fig. 5B, orange side chain) and His213 (Fig. 5B, pink side chain) make important contacts with the adenine rings via ionic and hydrogen bonds respectively [72]. Reflecting their likely importance for FAD binding, Asp210 and His213 and Lys217 are highly conserved (Fig. 5A black boxes).

The crystal structures for the human and bacterial MTHFR FAD binding pockets including both Ala222 and the α6 helix are highly superimposable [72, 73]. In fact, the Ala177Val variant in *E.coli* MTHFR (equivalent to Ala222Val in the human MTHFR) results in a displacement of the α5 helix (equivalent to the α6 helix in the human MTHFR) and disruption of the interactions with FAD (not shown) [74]. As a result, there is an increased propensity for the Ala177Val variant to dissociated from FAD which leads to a loss of enzyme activity due to instability of the apoenzyme [73, 75]. Together, these structural studies suggest that the Ala222Val polymorphism in human MTHFR leads to decreased FAD binding and enzyme instability.

Consistent with this notion, the Ala222Val substitution is associated with a ~ 30% reduction of total MTHFR catalytic activity recovered from homozygous 677TT individuals compared to homozygous wild-type individuals [76]. This reduced enzymatic activity does not appear to affect the enzyme specific activity, but instead leads to reduced conformational stability as the Ala222Val variant is more likely to dissociate into monomers and is significantly more thermolabile compared to the wild-type protein [72, 73, 76]. Thus, Ala222 likely makes contributions to FAD binding that are important for stabilizing the active conformation of the catalytic domain of MTHFR similar to the chemical chaperone-like roles proposed above for other flavoproteins.

Given the structural implications of the Ala222Val to FAD binding, it is striking that epidemiological evidence and Genomic Wide Association Studies (GWAS) suggest that the C677T polymorphism is associated with an increased risk of hypertension [77–79]. Furthermore, 677TT homozygous individuals with either low or deficient riboflavin have a threefold increased risk of hypertension [80]. Notably, clinical trials have reported that the C677T variant is associated with a riboflavin-responsive reduction in blood pressure (BP). For example, 1.6mg/day riboflavin treatment of clinically hypertensive individuals who were homozygous for the 677TT genotype and refractory to anti-hypertensives (beta-blockers and/or angiotensin-converting enzyme inhibitors) not only resulted in a significant reduction of their plasma homocysteine, but also reduced blood pressures to ≤ 140/90 mmHg in the majority of participants [81]. While these findings need to be replicated in larger trails and geographically diverse cohorts, they suggest that riboflavin supplementation could offer a genotype-specific treatment for the personalised management of hypertension in patients with the C677T variant. [80, 82, 83] Thus, it is possible that riboflavin therapy increasing FAD concentrations may stabilize the Ala222Val variant and thereby ameliorate adverse cardiovascular outcomes. Given that an estimated 1.13 billion people worldwide with hypertension have a significantly increased risk of developing cardiovascular diseases such as atherosclerosis, stroke and coronary heart disease, the prospective genetic identification of at-risk individuals with the Ala222Val variant for prophylactic riboflavin therapy may offer a safe non-drug approach for BP management with substantial longer-term health and economic benefits [84, 85].

## Mechanisms of riboflavin responsiveness in adult-onset disease

The precise molecular mechanisms underpinning the flavo-enzyme variants described herein that lead to the range of late-onset riboflavin-responsive pathologies remains unclear. Very few individuals who exhibit riboflavin-responsiveness demonstrate riboflavin deficiency [86, 87]. However, at least in part, FAD appears to function as a chemical chaperone that is important for correct flavo-enzyme folding and stability. In this respect, several studies have shown that depletion of riboflavin or FAD leads to the rapid degradation of several flavoenzymes [88, 89]. Thus, amino acid variants that reduce FAD binding and/or affinity would be proposed to destabilize the apo-enzyme which would then lead to loss of enzyme function and degradation. In line with this proposal, increasing riboflavin intake would lead to increased intracellular FAD concentrations that would shift the equilibrium toward FAD binding to the apo-enzyme thereby stabilizing tertiary protein structure. In some cases, the ability of increased concentrations of riboflavin to restore flavoprotein stability has been directly observed in vitro in cells derived from individuals expressing ETFDH, FLAD1, ACAD9 and MTHFR variants that exhibit riboflavin-responsive phenotypes [54, 60, 90, 91]. In addition, supplementation of FAD to MTHFR and EFT:QO in vitro has been shown to help rescue enzyme stability and activity following incubation at 46 °C and 40 °C respectively, particularly in variants known to be heat sensitive [54, 72]. The corollary of these in vitro observations in patients is that the expression of an unstable flavoprotein variant due to its reduced FAD binding might be reversed using high-dose riboflavin therapy that increases FAD concentrations to levels favouring apoprotein binding and stability.

Unlike most monogenic pathologies, the age of onset, spectrum of symptoms and their severity in adult-onset riboflavin-responsive diseases varies enormously. For example, while the median age of first symptoms in late-onset MADD associated with ETF-QO variants has been reported to be 19.2 years, in some cases, individuals experience the onset of symptoms in their sixth and seventh decades [86]. Furthermore, adult-onset riboflavin-responsive diseases frequently arise in individuals with no family history and from non-consanguineous parents. Thus, it appears that in addition to the specific SNPs that predispose individuals to adult-onset riboflavin-responsive diseases, there are likely additional genetic and/or environmental triggers.

For many individuals, the triggers for adult-onset riboflavin-responsive disease are not known. However, the identification of precipitating factors, at least in some cases, has provided insights into the possible molecular basis for adult-onset disease. For example, infections and febrile illnesses have been observed to immediately precede late-onset riboflavin-responsive illness associated with variants of ETF-QO, ETF and ACAD9 (Box 1) [48, 51, 92]. The catabolic stresses associated with infections are known to lead to decreased tissue riboflavin and FAD levels in both mice and humans [93]. Thus, in the context of flavoprotein variants with reduced FAD binding, it may be only after a specific catabolic stressor such as an infection where FAD concentrations fall below a critical threshold that there is no longer sufficient FAD to bind, stabilize and promote flavoprotein activity.

**Table:** 

**Box 1 Potential triggers for adult-onset riboflavin-responsive pathologies**
*Diet and lifestyle:*
• Restricted or suboptimal eating patterns of Vitamin B2-containing foods (eg. meat, fortified cereals and green vegetables)
• Fasting
• Exercise
*Life stage:*
• Pregnancy
• Midlife or older adults
*Medications*
• Metformin (diabetes)
• Triiodothyronine (thyroid hormone)
• Valproate, topiramate (epilepsy, bipolar disorder)
• Tenofovir, Alafenamide (antiviral)
*Health*
• Infections
• Fever
• Crohn, Celiac or inflammatory bowel diseases

In addition to infections, several adult-onset riboflavin-responsive pathologies associated with variants in riboflavin transporters, ETF-QO and FADS have been associated with pregnancy (Box 1) [39, 51, 90, 94]. For example, variants in riboflavin transporters that do not appear to have any pathologic consequences in non-pregnant females have been reported to lead to maternal pathology once pregnant [17, 18]. As is the case with infections, the catabolic demands of pregnancy are known to lead to increase in riboflavin requirements. [3, 95, 96] Other catabolic stressors likely increase riboflavin requirements such as fasting, exercise and cold exposure have also been associated with late-onset riboflavin-responsive disease (Box 1) [47, 51, 90, 92]. 

Intriguingly, a number of medications have been associated with adult-onset riboflavin-responsive diseases (Box 1). For example, drugs such as metformin, valproate and anti-viral drugs have been reported as inducing metabolic decompensation that can be at least partially mitigated by riboflavin therapy [97, 98]. Importantly, these drugs have been identified as inducing mitochondrial toxicity in some patients possibly leading to impaired utilization of FAD and oxidative phosphorylation [99, 100]. While the underlying mechanisms were not investigated in these reports, it is possible that the presence of SNPs within flavoenzyme hotspots predisposed the affected individuals to drug-induced perturbations in FAD levels leading to drug hypersensitivity.

The factors listed in Box 1 may act as metabolic or biochemical ‘tipping points’ to trigger symptoms in individuals harbouring flavoprotein variants with impaired FAD binding. Such a notion would be consistent with a liability threshold model in which a specific SNP in a flavoprotein confers a latent phenotype which, when exposed to one or more risk factors, leads to adult-onset disease. Investigation of such a liability threshold model may allow the development of a risk score that combines the genetic risk conferred by specific flavoprotein hotspot mutations with the environmental triggers shown in Box 1. In addition, genome-wide association studies (GWAS) may also provide important clues to possible gene variants that further increase risk when combined with SNPs within flavoprotein genes.

## Future perspectives

Owing to their diverse array of symptoms together with wide variation in age of onset, patients with riboflavin-responsive pathologies often experience lengthy delays in obtaining a diagnosis. In cases of adult-onset riboflavin-responsive diseases caused by ETFDH, FLAD1, SLC52A2, SLC52A3 and SLC25A32 mutations, the time from the onset of symptoms to diagnosis can take years, and sometimes even decades [101, 102]. More broadly, surveys involving individuals with a mitochondrial disease have reported that patients saw an average of 8.2 clinicians and the majority (54.6%) received one or more incorrect diagnoses before their final mitochondrial diagnosis [103].

In part, such diagnostic odysseys could be due to entrenched views that RDI settings for riboflavin should be adequate for all individuals irrespective of genotype. While the success of riboflavin therapy has been reported in adult-onset disease for over 80 years, such successes in the pre-genome era were not based on known flavoprotein networks and their functions (Fig. 1) and so were largely fortuitous [104]. However, the application of NGS approaches can now not only provide a rational approach to the diagnosis of a wide spectrum of adult-onset riboflavin-responsive diseases but also reduce the time to diagnosis. For example, whole exome sequencing of a cohort of patients with undiagnosed muscular, neuromuscular, and metabolic symptoms in which mitochondrial involvement was suspected was successful in identifying a disease-causing genetic defect in 68% of individuals [105]. Notably, a subset of these patients included adult-onset disease including novel variants in the SLC25A46 and ACAD8 flavoproteins.

As point-of-care NGS becomes more widespread, identifying variants in flavoproteins that involve the FAD-binding hotspots which are riboflavin-responsive as discussed herein has the potential to uncover a much wider population of patients living with undiagnosed and untreated chronic illnesses. Once a flavoprotein mutation has been identified in a patient, the structure:function relationships presented herein could be used to inform clinical management approaches including riboflavin therapy. Taking into consideration the suspected significant numbers of undiagnosed patients with riboflavin-responsive pathologies together the personal, economic and clinical burdens that accompany their long-term disability, a personalized ‘nutrigenomic’ approach in which patients identified with riboflavin-responsive mutations could be initiated with a low cost and safe riboflavin therapy.

### Supplementary Information

**Additional file 1**: The Flavoproteome—Proteins requiring riboflavin or flavin cofactors for their function.

## Data Availability

Not applicable.
