# Supplementary material for: New insights into the nutritional genomics of adult-onset riboflavin-responsive diseases
Source: Nutr Metab (Lond). 2023 Oct 16;20:42. doi: 10.1186/s12986-023-00764-x (PMC10580530; doi:10.1186/s12986-023-00764-x)
Supplement: Supplementary file 1 — Additional file 1: The Flavoproteome—Proteins requiring riboflavin or flavin cofactors for their function. [file 12986_2023_764_MOESM1_ESM.docx]

**Table S1:** *-* ***The Flavoproteome*** *– Proteins requiring riboflavin or flavin cofactors for their function.*

| **No.** | **Enzyme** | **Gene symbol** | **Gene location** | **Cofactor/ ligand** |
| --- | --- | --- | --- | --- |
| 1 | D-lactate dehydrogenase | LDHD | 16q23.1 | FAD |
| 2 | Xanthine dehydrogenase | XDH | 2p23.1 | FAD |
| 3 | (*S*)-2-hydroxy-acid oxidase | HAO1 | 20p12.3 | FMN |
| 4 | hydroxyacid oxidase 2 | HAO2 | 1p12 | FMN |
| 5 | Glycerol 3-phosphate dehydrogenase | GPD2 | 2q24.1 | FAD |
| 6 | Choline dehydrogenase | CHDH | 3p21.1 | FAD |
| 7 | L-2-Hydroxyglutarate dehydrogenase | L2HGDH | 14q21.3 | FAD |
| 8 | D-2-Hydroxyglutarate dehydrogenase | D2HGDH | 2q37.3 | FAD |
| 9 | Aldehyde oxidase | AOX1 | 2q33.1 | FAD |
| 10 | Dihydropyrimidine dehydrogenase | DPYD | 1p21.3 | FAD FMN |
| 11 | 3b-Hydroxysterol D^24^-reductase | DHCR24 | 1p32.3 | FAD |
| 12 | Dihydroorotate dehydrogenase | DHODH | 16q22.2 | FMN |
| 13 | Protoporphyrinogen IX oxidase | PPOX | 1q23.3 | FAD |
| 14 | Peroxisomal acyl-coenzyme A oxidase 1 | ACOX1 | 17q25.1 | FAD |
| 15 | Peroxisomal acyl-coenzyme A oxidase 2 | ACOX2 | 3p14.3 | FAD |
| 16 | Peroxisomal acyl-coenzyme A oxidase 3 | ACOX3 | 4p16.1 | FAD |
| 17 | Glutaryl-CoA oxidase | C7orf10 | 7p14.1 | FAD |
| 18 | Succinate dehydrogenase Flavoprotein subunit A | SDHA | 5p15.33 (3q29)a | FAD |
| 19 | Short-chain- (butyryl-) acyl CoA dehydrogenase | ACADS | 12q24.31 | FAD |
| 20 | Medium-chain acyl-CoA dehydrogenase | ACADM | 1p31.1 | FAD |
| 21 | Glutaryl-CoA dehydrogenase | GCDH | 19p13.2 | FAD |
| 22 | Isovaleryl-CoA dehydrogenase | IVD | 15q15.1 | FAD |
| 23 | 2-Methylbutyryl-CoA dehydrogenase | ACADSB | 10q26.13 | FAD |
| 24 | Long-chain-acyl-CoA dehydrogenase | ACADL | 2q34 | FAD |
| 25 | Very long-chain acyl-CoA dh | ACADVL | 17p13.1 | FAD |
| 26 | Isobutyryl-CoA dehydrogenase | ACAD8 | 11q25 | FAD |
| 27 | Complex I assembly factor ACAD9, mitochondrial | ACAD9 | 3q21.3 | FAD |
| 28 | Long- and branched-chain-acyl-CoA dh | ACAD10 | 12q24.1 | FAD |
| 29 | C22-long-chain-acyl-CoA dehydrogenase | ACAD11 | 3q22.1 | FAD |
| 30 | D-aspartate oxidase | DDO | 6q21 | FAD |
| 31 | L-amino acid oxidase | IL4I1_2 | 19q13.3-q13.4 | FAD |
| 32 | D-amino acid oxidase | DAO | 12q24.11 | FAD |
| 33 | Monoamine oxidase | MAOA | Xp11.3 | FAD |
| 34 | Pyridoxal 5’-phosphate oxidase | PNPO | 17q21.32 | FMN |
| 35 | Catecholamine oxidase (renalase) | RNLS | 10q23.31 | FAD |
| 36 | Methylenetetrahydrofolate reductase | MTHFR | 1p36.22 | FAD |
| 37 | L-pipecolate oxidase | PIPOX | 17q11.2 | FAD |
| 38 | Spermine oxidase | SMO | 20p13 | FAD |
| 39 | Electron-transferring ﬂavoprotein-ubiquinone oxidoreductase | ETFDH | 4q32.1 | FAD |
| 40 | Electron transferring ﬂavoprotein | ETFA | 15q24.2-q24.3 | FAD |
| 41 | Electron transferring ﬂavoprotein B | ETFB | 19q13.41 | FAD |
| 42 | Sarcosine dehydrogenase | SARDH | 9q34.2 | FAD |
| 43 | Dimethylglycine dehydrogenase | DMGDH | 5q14.1 | FAD |
| 44 | Lysine-specific histone demethylase 1A | KDM1A | 1p36.12 | FAD |
| 45 | Lysine-speciﬁc histone demethylaseB | KDM1B | 6p22.3 | FAD |
| 46 | Proline dehydrogenase | PRODH | 22q11.21 | FAD |
| 47 | Cytochrome-b5 reductase | CYB5R3 | 22q13.2 | FAD |
| 48 | NADPH-hemoprotein reductase (cytochrome P450 reductase) | POR | 7q11.23 | FAD |
| 49 | NAD(P)H dehydrogenase (quinone) | NQO1 | 16q22.1 | FAD |
| 50 | NADH-ubiquinone oxidoreductase of complex I, subunit UQOR1 | NDUFV1 | 11q13.2 | FMN |
| 51 | NADPH-dep. diﬂavin oxidoreductase 1 | NDOR1 | 9q34.3 | FAD FMN |
| 52 | tRNA dihydrouridine synthase | DUS2L | 16q22.1 | FMN |
| 53 | Dihydrolipoyl dehydrogenase | DLD | 7q31.1 | FAD |
| 54 | Glutathione-disulﬁde reductase | GSR | 8p12 | FAD |
| 55 | Thioredoxin-disulﬁde reductase 1 | TXNRD1 | 12q23.3 | FAD |
| 56 | Thioredoxin-disulﬁde reductase 2 | TXNRD2 | 22q11.21 | FAD |
| 57 | Thioredoxin-disulﬁde reductase 3 | TXNRD3 | 3q21.3 | FAD |
| 58 | ER ﬂavoprotein associated with degr. | FOXRED2 | 22q12.3 | FAD |
| 59 | Sulfhydryl oxidase | GFER | 16p13.3 | FAD |
| 60 | Prenylcysteine oxidase | PCYOX1 | 2p13.3 | FAD |
| 61 | Ribosyldihydronicotinamide dehydrogenase | NQO2 | 6p25.2 | FAD |
| 62 | Flavin-containing monooxygenases 1 | FMO1 | 1q24.3 | FAD |
| 63 | Flavin-containing monooxygenases 2 | FMO2 | 1q24.3 | FAD |
| 64 | Flavin-containing monooxygenases 3 | FMO3 | 1q24.3 | FAD |
| 65 | Flavin-containing monooxygenases 4 | FMO4 | 1q24.3 | FAD |
| 66 | Flavin-containing monooxygenases 5 | FMO5 | 1q21.1 | FAD |
| 67 | Kynurenine 3-monooxygenase | KMO | 1q43 | FAD |
| 68 | Nitric-oxide synthase | NOS1 | 12q24.22 | FAD FMN |
| 69 | Nitric-oxide synthase | NOS2 | 17q11.2 | FAD FMN |
| 70 | Nitric-oxide synthase | NOS3 | 7q36.1 | FAD FMN |
| 71 | Squalene epoxidase | SQLE | 8q24.13 | FAD |
| 72 | Monooxygenase in coenzyme Q biosyn. | COQ6 | 14q24.3 | FAD |
| 73 | Ferrireductase (biliverdin IX beta red.) | STEAP3 | 2q14.2 | FAD |
| 74 | Methionine synthase reductase | MTRR | 5p15.31 | FAD FMN |
| 75 | Ferredoxin-NADP^+^ reductase | FDXR | 17q25.1 | FAD |
| 76 | NAD(P)H oxidase cytochrome b(558), beta subunit | CYBB | Xp11.4 | FAD |
| 77 | Thyroid oxidase / dual oxidase | DUOX1 | 15q21.1 | FAD |
| 78 | Acetolactate synthase-like protein | ILVBL | 19p13.12 | FAD |
| 79 | Alkyldihydroxyacetone phosphate synthase | AGPS | 2q31.2 | FAD |
| 80 | 4’-Phosphopantothenoylcysteine decarboxylase | PPCDC | 15q24.2 | FMN |
| 81 | Cryptochrome | CRY1 | 12q23.3 | FAD |
| 82 | Cryptochrome | CRY2 | 11p11.2 | FAD |
| 83 | Apoptosis inducing protein | AIFM1 | Xq26.1 | FAD |
| 84 | Apoptosis inducing protein | AIFM2 | 10q22.1 | FAD |
| 85 | Iodotyrosine deiodinase | IYD | 6q25.1 | FMN |
| 86 | Microtubule associated monooxygenase1 | MICAL1 | 6q21 | FAD |
| 87 | Microtubule associated monooxygenase2 | MICAL2 | 11p15.3 | FAD |
| 88 | Microtubule associated monooxygenase3 | MICAL3 | 22q11.21 | FAD |
| 89 | FAD-dependent oxidoreductase (molecular chaperone of complex 1) | FOXRED1 | 11q24.2 | FAD |
| 90 | Riboﬂavin transporter 1 | SLC52A1 | 17p13.2 | Riboﬂavin |
| 91 | Riboﬂavin transporter 2 | SLC52A2 | 8q24.3 | Riboﬂavin |
| 92 | Riboﬂavin transporter 3 | SLC52A3 | 20p13 | Riboﬂavin |
| 93 | Riboﬂavin / FMN reductase | BLVRB | 19q13.2 | Riboﬂavin |
| 94 | Riboﬂavin kinase | RFK | 9q21.13 | Riboﬂavin |
| 95 | FAD-adenylyl transferase (synthetase) | FLAD1 | 1q21.3 | FMN |
| 96 | Solute carrier family 25 member 32 | SLC25A32 | 8q22.3 | Riboﬂavin |

**Supplementary Table S1 legend**

*List of proteins requiring riboflavin or flavin cofactors for their function {Lienhart, 2013 #4} Uniprot {Coudert, 2023 #457} there are 6 proteins binding both FMN and FAD, 75 requiring FAD and 9 FMN; the remaining 6 proteins are* *riboflavin transporters (90-92 and 96) and cytosolic enzymes (93 and 94).*
